# CARING SELF-EFFICACY AND BEHAVIORS OF MIGRANT NURSING ASSISTANTS WORKING IN RESIDENTIAL AGED CARE FACILITIES

**DOI:** 10.1093/geroni/igad104.1876

**Published:** 2023-12-21

**Authors:** Sumina Shrestha, Christine While

**Affiliations:** La Trobe University, Bundoora, Victoria, Australia; La Trobe University, Bundoora, Victoria, Australia

## Abstract

Data show a significant proportion of nursing assistants from migrant backgrounds in the Australian aged care industry. However, compared to locally-born counterparts, they are more likely to be employed as casual staff and offered shorter shifts, irregular hours, and lower pay, often without employment benefits. Previous studies have shown that the working environment of an organization influences employees’ self-efficacy, affecting their performance. This study hypothesized that migrant nursing assistants from non-English-speaking countries have lower confidence in caring for older residents and, thus, execute less-satisfactory caring behaviors than their local, English-speaking colleagues. The caring self-efficacy was quantitatively measured from the perspective of nursing assistants in residential aged care facilities (N = 280), and caring behaviors were explored from older residents’ perspective (N = 13). Results showed that nursing assistants whose first language was not English had lower levels of caring self-efficacy than native English speakers. However, older residents found that they frequently met residents’ individual needs and were more competent, emotionally present, respectful, and amiable, despite communication issues. Older residents insisted that the personalities of nursing assistants have a greater impact on their job performance than their backgrounds. These findings suggest that although migrant nursing assistants from non-English-speaking countries had low self-efficacy to carry out their job, their performance was appreciated by older residents. This study supports the Australian government’s strategy of using immigration to prevent or respond to an aged care workforce crisis, but it also advocates for improved working conditions, organizational resources and benefits for those with non-English-speaking backgrounds.

